# The Role of Circulating MicroRNA-126 (miR-126): A Novel Biomarker for Screening Prediabetes and Newly Diagnosed Type 2 Diabetes Mellitus

**DOI:** 10.3390/ijms150610567

**Published:** 2014-06-12

**Authors:** Yang Liu, Guangqiang Gao, Chun Yang, Kun Zhou, Baozhong Shen, Hongyan Liang, Xiaofeng Jiang

**Affiliations:** 1 Department of Clinical Biochemistry Laboratory, the 4th Affiliated Hospital of Harbin Medical University, #37 Yiyuan Street, Nangang District, Harbin 150001, China; E-Mails: yl15904506198@163.com (Y.L.); ggqsyjyk@163.com (G.G.); ycsyjyk@126.com (C.Y.); zksyjyk@126.com (K.Z.); 2 Heilongjiang Province Key Laboratory of Molecular Image, Harbin 150001, China; E-Mail: sbzsyyx@163.com

**Keywords:** microRNA-126, biomarker, IGT/IFG, pre-diabetes, type 2 diabetes mellitus

## Abstract

Recent studies suggested an association of endothelial microRNA-126 (miR-126) with type 2 diabetes mellitus (T2DM). In the current study, we examined whether circulating miR-126 is associated with T2DM and pre-diabetic syndrome. The study included 82 subjects with impaired glucose tolerance (IGT), 75 subjects with impaired fasting glucose (IFG), 160 patients with newly diagnosed T2DM, and 138 healthy individuals. Quantitative polymerase chain reaction (qPCR) was used to examine serum miR-126. Serum miR-126 was significantly lower in IGT/IFG subjects and T2DM patients than in healthy controls (*p* < 0.05). After six months of treatment (diet control and exercise in IGT/IFG subjects, insulin plus diet control and exercise in T2DM patients), serum miR-126 increased significantly (*p* < 0.05). An analysis based on serum miR-126 in the sample revealed a significantly higher odds ratio (OR) for the subjects with the lowest 1/3 of serum miR-126 for T2DM (OR: 3.500, 95% confidence interval: 1.901–6.445, *p* < 0.05) than subjects within the highest 1/3 of serum miR-126. Such an association was still apparent after adjusting for other major risk factors. The area under the curve (AUC) for the receiver-operating characteristic (ROC) analysis was 0.792 (95% confidence interval: 0.707–0.877, *p* < 0.001). These results encourage the use of serum miR-126 as a biomarker for pre-diabetes and diabetes mellitus, as well as therapeutic response.

## 1. Introduction

MicroRNAs (miRNAs) are endogenous small noncoding RNAs of 21–25 nucleotides that could bind to 3' untranslated region of the mRNAs of protein-coding genes to down-regulate their expression [1,2]. MiRNAs play important roles in cellular proliferation, apoptosis, and differentiation [3,4,5]. MiRNAs could be detected in plasma and serum [6] as well as a variety of other fluids, including saliva and urine [7,8]. Changes of individual miRNAs and the miRNA signatures are linked to the diagnosis and prognosis of various diseases [9,10]. Tumor-specific miRNAs have been identified in cancer patients [11]. Tissue-derived plasma miRNAs have been used as biomarkers for injury [12,13,14]. In addition, changes of circulating miRNAs have been found in cardiovascular diseases, including myocardial infarction, coronary artery disease (CAD) and heart failure, as well as autoimmune diseases [14,15,16,17,18,19].

Type 2 diabetes mellitus (T2DM) is a risk factor for a variety of cardiovascular diseases, primarily through micro- and macro-vascular complications [20,21]. As a matter of fact, in T2DM patients, CAD is a major cause of death [22].

A population-based cohort study identified a strong association of miR-126 with T2DM [23]. miR-126 is highly enriched in endothelial cells, and contributes to the maintenance and repair of vascular integrity, angiogenesis, and wound repair [24,25]. In the present study, we examined serum miR-126 in subjects with impaired glucose tolerance (IGT) or impaired fasting glucose (IFG), patients with newly diagnosed T2DM *vs.* healthy control subjects.

## 2. Results and Discussion

### 2.1. Detection of Cel-miR-39 as a Negative Control

In validation experiments with exogenous cel-miR-39, we did not find a significant difference of serum cel-miR-39 between T2DM patients and healthy controls (20.18 ± 0.32 *vs.* 20.36 ± 0.29, *n* = 30, *p* = 0.549), suggesting that the use of serum from T2DM patients does not introduce systematic errors in reverse transcription and PCR.

### 2.2. Baseline Characteristics and Serum miR-126 vs. Age/Gender

Major characteristics of the subjects are listed in Table 1. Serum miR-126 did not differ significantly between those at ≤ and >50 years of age, and between men and women (both *p* > 0.05; Table 2).

**Table 1:** Clinical characteristics for patients of impaired glucose tolerance (IGT)/impaired fasting glucose (IFG), diabetes mellitus (DM) and healthy controls.

Characteristics	Healthy Controls (138 *)	IGT/IFG (157)		DM (160)		P1	P2	P3
		Before Treatment	After Treatment	Before Treatment	After Treatment			
Sex (M:F)	67/71	82/75		78/82		0.793		
Age (year)	46.7 ± 7.2	47.9 ± 7.8		50.2 ± 6.7		0.985		
BMI (kg/m^2^)	22.87 ± 0.32	23.10 ± 0.26		23.32 ± 0.31		0.314		
Glu (mmol/L)	4.54 ± 0.66	5.13 ± 0.62	4.64 ± 0.57	11.20 ± 2.65	8.14 ± 2.42	<0.001	<0.05	<0.05
HbAlc (%)	4.69 ± 0.57	6.25 ± 0.67	5.62 ± 0.45	9.16 ± 1.64	7.02 ± 1.84	<0.001	<0.05	<0.05
CH	3.87 ± 0.74	4.12 ± 0.86	4.02 ± 0.54	4.99 ± 1.52	4.68 ± 1.48	0.253	0.462	0.543
TG	1.43 ± 0.56	1.51 ± 0.43	1.48 ± 0.37	2.43 ± 3.3	2.23 ± 2.4	0.461	0.532	0.732
HDL	1.54 ± 0.22	1.53 ± 0.18	1.54 ± 0.16	1.49 ± 0.16	1.47 ± 0.20	0.375	0.422	0.653
LDL	2.35 ± 0.52	2.51 ± 0.47	2.50 ± 0.32	2.46 ± 0.38	2.44 ± 0.41	0.422	0.674	0.782
Lp (a)	14.53 ± 8.48	17.86 ± 7.52	16.64 ± 7.36	16.54 ± 7.23	17.04 ± 8.54	0.324	0.486	0.529
miR-126 [lg 2^(50–*C*t)^]	8.58 ± 0.62	7.70 ± 0.41	7.99 ± 0.12	6.17 ± 0.57	6.63 ± 0.62	<0.05	0.036	0.001

* Indicates numbers of subjects in the group. P1: comparison to healthy controls, IFG/IGT and newly diagnosed type 2 diabetes mellitus; P2: comparison between IFG/IGT patients before treatment *vs.* after treatment; P3: comparison between DM patients before treatment * vs.* after treatment; IFG, impaired fasting glucose; IGT, impaired glucose tolerance; DM, diabetes mellitus; BMI, body mass index; HbAlc, glycosylated hemoglobin; CH, cholesterol; TG, triglyceride; HDL, high density lipoproteins; LDL, low density lipoproteins; Lp (a), lipoprotein (a).

**Table 2:** The distribution of serum miR-126 in different groups.

Variables	Control			IGT/IFG			DM Patients		
	Number	miR-126 Median (95% CI)	*p* Value	Number	miR-126 Median	*p* Value	Number	miR-126 Median	*p* Value
Age (years)									
≤50	68	8.79 (8.33–9.02)	0.565	72	7.62 (7.36−7.94)	0.209	78	6.32 (5.96−6.45)	0.897
>50	70	8.62 (8.40–8.95)	–	85	7.96 (7.66−8.10)	–	82	6.18 (5.78−6.51)	–
Gender									
Male	73	8.60 (8.41–8.88)	0.565	79	7.96 (7.62−8.09)	0.337	77	7.81 (8.37−8.04)	0.553
Female	65	8.74 (8.33–8.96)	–	78	7.66 (7.41–7.98)	–	83	6.33 (5.86–6.46)	–

### 2.3. Serum miR-126 across Disease Conditions

The reverse transcription quantitative polymerase chain reaction (qRT-qPCR) analysis demonstrated significantly higher *C*_t_ values (presented as median and interquartile range) of miR-126 at 24.32 (23.38–25.45) in IGT/IFG subjects and 29.34 (28.35–30.93) in T2DM patients *vs.* 21.38 (19.82–22.62) in healthy donors (*p* < 0.001).

**Figure 1:**
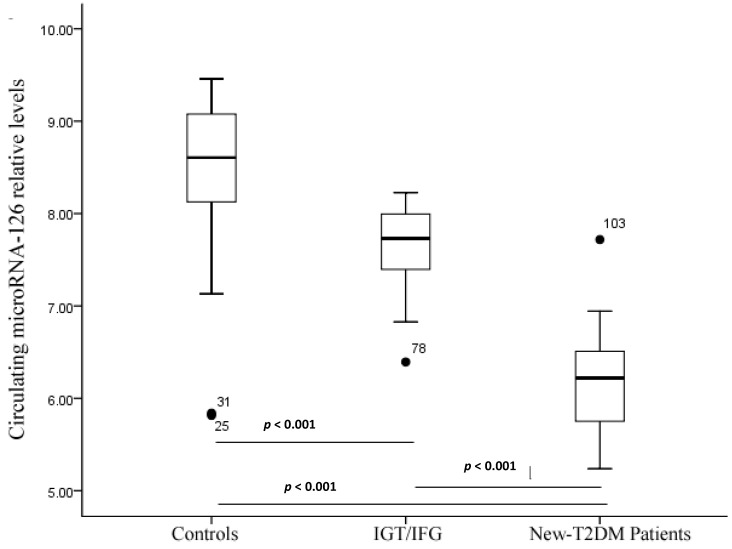
Circulating miR-126 levels in the control, IGT/IFG, and new-T2DM patients. Data as shown as median (interquartile range). *p* < 0.001 compared to controls. Abbreviations: new-T2DM, newly diagnosed type 2 diabetes mellitus.

Serum miR-126 concentration was significantly lower in IGT/IFG and T2DM patients than in healthy controls (Figure 1). Also, serum miR-126 concentration was significantly lower in T2DM patients than in the IGT/IFG subjects (*p* < 0.001).

### 2.4. Treatment Response

**Figure 2:**
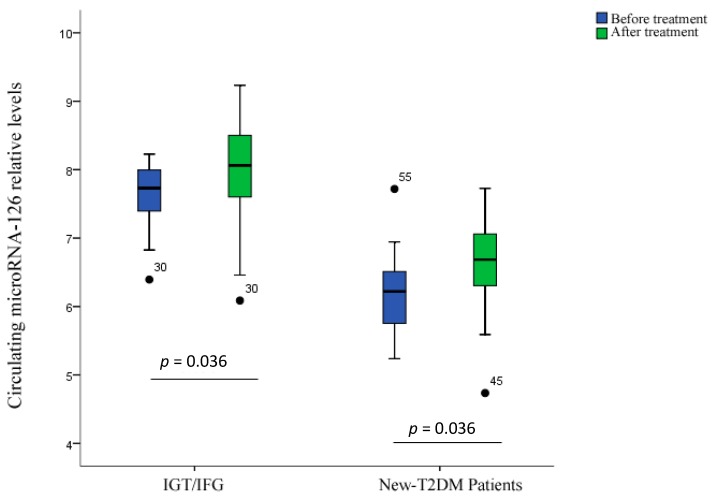
Changes in miR-126 levels after six months of treatment. Circulating miR-126 levels in IGT/IFG, new-T2DM patients before and after treatment. Data are shown as median (interquartile range).

Diet control and physical exercise in IGT/IFG subjects for six months increased serum miR-126 level (*p* = 0.036 *vs.* pretreatment baseline; Figure 2). Treatment in T2DM patients (insulin plus diet control and exercise for six months) also increased serum miR-126 level (*p* = 0.001).

### 2.5. Correlation Analysis

In this analysis, we divided the subjects into three subgroups based on serum miR-126 (with lowest, middle, and highest concentration), and examined the odds ratio (OR) for T2DM (Table 3). Higher OR (3.500 relative to those with highest 1/3 miR-126, 95% confidence interval (CI), 1.901–6.445, *p* < 0.05) for T2DM, was noticed in the subjects with serum miR-126 in the lowest 1/3. Adjusting for age, gender, body mass index (BMI) and some biochemical indicators did not substantially change the results.

**Table 3:** Odds ratios (95% CI) for new-T2DM, by tertiles of circulating miR-126 levels.

Variable	Tertiles of Serum miR-126 Levels			*p*-Trend
	1 (Lowest)	2	3 (Highest)	
MiR-126 levels	≤8.32	8.32–8.82	≥8.82	
New-T2DM patients/Controls, *n*/*n*	84/46	52/46	24/46	<0.05
Crude OR (95% CI)	3.500 (1.901–6.445)	1.615 (0.946–2.759)	1	<0.05
Adjusted OR (95% CI), Model	3.825 (2.336–8.547)	1.834 (0.952–3.014)	1	<0.05

**Figure 3:**
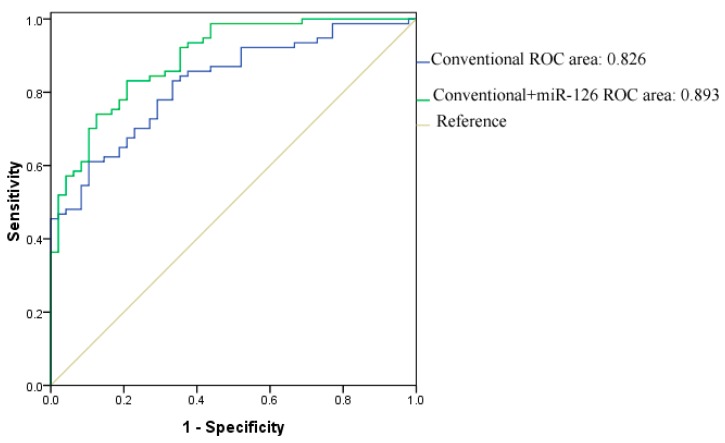
ROC curves and corresponding AUCs for new-T2DM. The AUC in an established conventional model was 0.826 (95% CI, 0.756–0.897). After circulating miR-126 levels were introduced, the AUC was 0.893 (95% CI, 0.838–0.947). Abbreviations: AUC, area under the curve; CI, confidence interval; ROC, receiver operating characteristic.

In a receiver operator characteristic (ROC) analysis, using miR-126 alone, distinguished T2DM patients from the healthy controls with an area under the curve (AUC) at 0.792 (95% CI, 0.707–0.877, *p* < 0.001, Figure 3). The AUC ROC analysis that included age, gender, BMI, blood glucose, HbAlc but not miR-126 was 0.826 (95% CI, 0.756–0.897) for new-T2DM. Adding miR-126 to the analysis increased the AUC to 0.893 (0.838–0.947, *p* < 0.05 *vs.* the AUC in the analysis without miR-126).

Previous studies have indicated an association between miR-126 and T2DM [23]. The results from the current study extended the association to pre-diabetes (e.g., IFG/IGT subjects).

miR-126 is highly enriched in endothelial cells, and plays a pivotal role in maintaining endothelial homeostasis and vascular integrity [25]. It represses two negative regulators of the vascular endothelial growth factor (VEGF) pathway (Sprouty-related protein (SPRED1) and phosphoinositol-3 kinase regulatory subunit 2 (PIK3R2/p85-β)), with an end result of enhanced VEGF signaling [24]. A previous study revealed that shedding miR-126 from endothelial cells could regulate VEGF responsiveness and confer vascular protection in a paracrine manner [26,27].

In this study, a RT-qPCR direct serum assay (RT-qPCR-DS) was used to examine serum miR-126. This assay eliminates the RNA extraction step, and therefore, could avoid miRNA loss, minimize human and mechanical errors, and reduce the time and overall cost [28]. Analysis of miR-126 transcripts obtained from the serum of T2DM patients revealed a correlation between T2DM and miR-126 expression. The current study indicated that serum miR-126 could be used to discriminate pre-diabetes from healthy individuals. Also, low miR-126 expression was significantly associated with poor treatment response.

ROC analysis showed reasonable sensitivity and specificity of serum miR-126 in distinguishing IFG/IGT and T2DM patients from healthy individuals.

In the current study, we also noticed an association of decreased serum miR-126 with high blood glucose; accordingly, the miR-126 content in plasma was reduced in a glucose-dependent fashion. This association suggests that elevated plasma glucose might result in the reduced delivery of miR-126 to monocytes, which in turn contributes to VEGF resistance and endothelial dysfunction.

Serum miR-126 has been linked with numerous diseases, including cancer. However, serum miR-126 has been reported to be both increased and decreased, depending on the type of cancer. miR-126 regulates the expression of several proteins with prominent roles in multiple diseases, including the anti-inflammatory TOM1 (target of Myb1), the growth factor VEGF-A, and the cell cycle regulatory and signaling protein IRS-1 (insulin receptor substrate 1). Based on the current study, we speculate that miR-126 secretion could be stimulated by insulin and suppressed by high blood glucose.

The sample size of the current study is relatively small. As a result, the findings should be considered preliminary and require further studies, ideally together with other molecular markers. Also, there is a lack of generally accepted standards for qRT-qPCR in body fluids. It is possible that *caenorhabditis elegans miRNA (C. elegans)* is spiked in control Minas [29]. We do, however, believe that no currently available methods are ideal. Since the factors remain unknown that could lead to biological variation independent of the disease being studied, and the effect on specific miRNAs have not been characterized, the use of any endogenous controls could introduce significant bias. The use of “invariant” miRNAs as endogenous controls has been proposed by some investigators, but tends not to affect the data significantly. Also, such a practice may not adjust for true biological variability since the “invariant” miRNAs may not be truly invariant. In our experiment, we did not use an internal control, producing further limitations. Nevertheless, we used identical amounts of serum for the assay, as reported earlier [30,31]. We understand that such a method is not commonly used, and may introduce technical problems. In future work, we will consider using multiple controls (for example, both external and several internal miRNAs control.

In conclusion, our results suggested that circulating miR-126 could be used to distinguish T2DM patients, as well as pre-diabetic subjects from healthy subjects. Further studies with a large cohort of patients are required to validate and develop miR-126 as a serum biomarker for T2DM and pre-diabetes, and possibly for monitoring disease progression and treatment response.

## 3. Experimental Section

### 3.1. Clinical Samples

Serum samples were collected from a total of 455 subjects, including 82 subjects with IGT, 75 subjects with IFG, 160 patients with newly diagnosed T2DM, and 138 healthy individuals. All subjects were recruited from the Department of Endocrinology in the Hospital of Harbin Medical University. Individuals with any other microvascular diseases, hypertension, coronary disease, systemic inflammatory diseases, acute respiratory infection, or cancers were excluded. The presence of T2DM was confirmed according to the World Health Organization criteria (WHO) [32]. No subjects received any treatment for T2DM or pre-diabetes prior to this study. The study was approved by the Ethics Committee of the 4th Affiliated Hospital of Harbin Medical University in October 2011. Written informed consent was obtained from all participants.

Blood samples were collected in tiger-top gel separator tubes (Thermo Fisher Scientific Inc., New York, NY, USA) in the morning after an overnight fasting. All samples were processed within 2–6 h of collection to obtain serum with centrifugation (12,000 rpm for 10 min) followed by filtering through a 13-mm serum filter (Thermo Fisher Scientific) and storage at −80 °C.

### 3.2. Diagnosis

The diagnosis of T2DM was established based on the guidelines by the World Health Organization (WHO) [32]. Normal glucose tolerance was defined as fasting glucose <110 mg/dL and 2 h glucose <140 mg/dL. IGT/IFG was defined as fasting glucose <126/140 mg/dL and 2 h glucose <200 mg/dL).

### 3.3. Reverse Transcription-Quantitative Polymerase Chain Reaction (RT-qPCR)

RT-qPCR-DS was performed using serum samples as described previously [28]. To deactivate or solubilize proteins, 2.5 μL serum sample was mixed with 2.5 μL reparation buffer containing 2.5% (*v*/*v*) Tween 20 (EMD Chemicals, Gibbstown, NJ, USA), 50 mmol/L Tris (Sigma-Aldrich, Saint Louis, MO, USA), and 1 mmol/L EDTA (Sigma-Aldrich). For RT reactions, 2.5 μL serum and equivalent preparation buffer mixture, 11.5 μL diethylpyrocarbonate, and 2 μL miR-126 RT primer (Applied Biosystems, Grand Island, NY, USA) were incubated at 70 °C for 10 min. Next, 5× buffer (5 μL), 10 mM dNTP (1 μL), and M-MLV reverse transcriptase (0.5 μL; Thermo Fisher Scientific Inc., Shanghai, China) were added, and the samples were incubated at 42 °C for 1 h, followed by 3 min at 93 °C for enzyme inactivation. The resulting cDNA was separated with centrifugation at 12,000× *g* for 10 min. The supernatant (2.0 μL) was used as the template for qPCR.

miR-126 was amplified using a TaqMan MicroRNA RT kit (Applied Biosystems), detected using TaqMan TM MicroRNA hsa-miR-126-specific primers (Applied Biosystems) quantified using the formula 2^(50−*C*t)^. Data were log transformed for analysis. The cycling conditions were: initial denaturation at 95 °C for 2 min, followed by 35 cycles of 95 °C for 10 s, 57 °C for 20 s, and 7 °C for 10 s. PCR was performed using a LightCycler TM 480 II System (Roche Applied Science, Basel, Switzerland) with the LightCycler 480 Probes Master kit (Roche Applied Science).

In order to examine whether serum condition affects the efficacy of reverse transcription and PCR, we carried out a validation experiment by adding exogenous cel-miR-39 to the assay. For RT reactions, 2.5 μL serum sample was mixed with 25 fmol (total volume 5 μL) synthetic *C. elegans* miRNAs cel-miR-39 (Qiagen, Valencia, CA, USA) (5'-UCACCGGGUGUAAAUCAGCUUG-3') and 2.5 μL of reparation buffer containing 2.5% (*v*/*v*) Tween 20 (EMD Chemicals), 50 mmol/L Tris (Sigma-Aldrich), and 1 mmol/L EDTA (Sigma-Aldrich). Samples were incubated for 5 min at room temperature. Then 2 μL cel-miR-39 RT primer (Qiagen) and 6.5 μL diethylpyrocarbonate were added and incubated at 70 °C for 10 min. Next, 5× buffer (5 μL), 10 mM dNTP (1 μL), and M-MLV reverse transcriptase (0.5 μL; Thermo Fisher Scientific) were added, and the samples were incubated at 42 °C for 1 h, followed by 3 min at 93 °C for enzyme inactivation. The resulting cDNA solution (supernatant) was obtained by centrifugation at 12,000× *g* for 10 min, and used as the template for qPCR.

Cel-miR-39 was amplified using a TaqMan MicroRNA RT kit (Qiagen), detected using TaqMan TM MicroRNA cel-miR-39 (Qiagen). The cycling conditions were: initial denaturation at 95 °C for 2 min, followed by 35 cycles of 95 °C for 10 s, 57 °C for 20 s, and 7 °C for 10 s. PCR was performed using a LightCycler TM 480 II System (Roche Applied Science, Penzberg, Germany) with the LightCycler 480 Probes Master kit (Roche Applied Science).

### 3.4. Statistical Analysis

Comparisons between cases and controls were performed using Chi-squared and Student’s *t*-test. Statistical significance was set at *p* < 0.05. Multivariate logistic regression was used to assess the association of T2DM with circulating miR-126. For OR and 95% CI for T2DM, circulating miR-126 level was divided into the lowest, middle and highest 1/3, based on the distribution in the healthy control group. OR was adjusted for known T2DM risk factors including age, gender, BMI and some biochemical indicators. Receiver operating characteristic (ROC) curve was used to estimate the diagnostic accuracy of each parameter, and the area under the curve (AUC) was reported. Statistical analyses were performed using SPSS software version 16.0 (SPSS Inc., Chicago, IL, USA). All statistical analyses were two-sided.

## 4. Conclusions

Serum miR-126 could be used as a biomarker for pre-diabetes and T2DM.
